# A Clue on the Skin: A Systematic Review on Immunohistochemical Analyses of the Ligature Mark

**DOI:** 10.3390/ijerph19042035

**Published:** 2022-02-11

**Authors:** Gelsomina Mansueto, Alessandro Feola, Pierluca Zangani, Antonietta Porzio, Anna Carfora, Carlo Pietro Campobasso

**Affiliations:** 1 Department of Advanced Medical and Surgical Sciences, University of Campania “Luigi Vanvitelli”, 80138 Naples, Italy; 2 Clinical Department of Laboratory Services and Public Health-Legal Medicine Unit, University of Campania “Luigi Vanvitelli”, Via Luciano Armanni 5, 80138 Naples, Italy; 3 Legal Medicine Unit, Department of Experimental Medicine, University of Campania “Luigi Vanvitelli”, Via Luciano Armanni 5, 80138 Naples, Italy; alessandro.feola@unicampania.it (A.F.); pierluca.zangani@unicampania.it (P.Z.); antonietta.porzio@studenti.unicampania.it (A.P.); anna.carfora@unicampania.it (A.C.); carlopietro.campobasso@unicampania.it (C.P.C.)

**Keywords:** ligature mark, skin, hanging, strangulation, immunohistochemistry

## Abstract

Background: A ligature mark is a common injury in cases of hanging or strangulation. Estimation of age and vitality of the ligature mark can be crucial for differentiating antemortem and postmortem wounds and to distinguish between simulated suicidal hanging or accidental strangulation to conceal a crime and not simulated events. The immunohistochemistry has been recommended by several Authors as a reliable tool to determine whether an injury was sustained during life or not. Unfortunately, no general agreement on the immunohistochemical markers to be used has been found among the scientific community. The aim of the study was to detect the type and function of the immunohistochemical markers useful in the assessment of the vitality and age of the ligature marks for routine diagnostics. Methods: Papers available on Pubmed, Scopus, and Web of Science were reviewed according to the PRISMA statement. Results: Only eight papers satisfied all the following inclusion criteria: full texts in English dealing with human ligature marks and immunohistochemistry published on impacted or indexed scientific journals. Conclusions: The assessment of the vitality of a ligature mark is still a challenging topic in forensic science. Under ideal conditions and in compliance with autopsy protocols, the diagnosis of death by hanging or strangulation on fresh bodies can be better supported by autopsy findings other than a ligature mark. The validation of immunohistochemical markers on large series could be of help in doubtful cases and differential diagnoses.

## 1. Introduction

A ligature mark is a well-known pressure mark on the neck underneath a ligature. It is a common injury in cases of hanging and strangulation, and sometimes, it is the only one recognized at autopsy. Except for ligature marks, often in these cases, there are no other external marks or internal injuries. The appearance of a ligature mark on the neck is subject to considerable variation, mostly depending on the nature and texture of the ligature [1]. A wide variety of tools, including electrical or telephone cords, ropes, wires, belts, ties, etc. can be used for producing a soft or hard ligature mark on the neck [1,2]. If the ligature is a soft material, such as a towel, the groove might be faint and pale, barely visible, poorly defined, and devoid of bruises and abrasions. If the ligature is a hard material, the furrow is usually recognizable and distinct, deep, well-demarcated, often with a mirror-image impression of the twist of the rope on the skin [1].

In hanging, the ligature mark generally does not completely encircle the neck, and it is deepest opposite the point of suspension because the noose commonly consists of a single loop. The corresponding furrow is usually located above the larynx, running obliquely, discontinuous towards the suspension point in an inverted V configuration. In ligature strangulation, in contrast to hangings, the ligature mark completely encircles the neck in a horizontal plane often overlying the larynx or upper trachea. Therefore, it is continuous and homogeneous, since the ligature is wrapped all around the neck surface and tightened by an external force to produce neck compression. Rarely, scratch marks can be seen above and below the ligature mark, although they can occur when the victim tries to remove the ligature or relieve the pressure or when the assailant places his hands around the ligature.

The determination of a vital reaction of human skin wounds is still a challenge for forensic pathologists. In forensic medicine, the term “vital reaction” describes the complex physiological events that occur at a systemic and local level when an extraneous force or noxa came into contact with a living organism [3]. Macroscopical and microscopical reactions of body tissues can be useful signs for differentiating antemortem and postmortem injuries and to determine whether an injury was sustained during life or not. Unfortunately, although extravasations of red blood cells and hemoglobin into the soft tissues surrounding the wounds are commonly considered a local reaction of vitality [4], caution has been recommended in the interpretation of such findings alone as vital signs [5]. Sometimes, even the gross hemorrhages, along with systemic vital reactions like cyanosis, oedema, or vascular congestion, cannot be identified [6], especially in badly decomposed or exhumed bodies.

One vitality marker alone is often of limited value, since it may be partly explained by resuscitation attempts or events that occurred during the supra-vital period [7]. Additional manifestations of local vital reactions at the site of injury, such as the beginning of the inflammation process with the immigration of polymorphonuclear leucocytes, require more time than is commonly available in traumatic deaths such as hanging and ligature strangulation [7]. In these cases, the survival time is often very short, as death mostly occurs within minutes [8], which is a not enough time for producing vital signs, such as the beginning of the wound healing process with the common inflammatory response [3,9,10]. This is the reason why the determination of vitality is closely related to the age estimation of skin wounds. Experts must be aware that several local and exogenous factors can affect the time course and appearance of local vital reactions after skin damage, among which are the manner and intensity of trauma and severity and extension of the alteration, temperature, pharmaceuticals, etc. [7]. The immunohistochemistry has been recommended by several Authors as a reliable tool in forensic pathology [11,12] and also to determine whether a human skin wound was sustained during life or not. Unfortunately, no validated immunohistochemical markers exist that can really help in solving the problem of the vitality and age estimation of a skin wound such as a ligature mark. No general agreement on the immunohistochemical markers to be used has been found among the scientific community. The aim of this study work was to perform a systematic review in order to detect the type and function of the immunohistochemical markers useful to distinguish, with certainty, vital skin lesions from ligature marks occurred after death or, better, in other words, useful in the assessment of vitality and age of the ligature marks in routine diagnostics.

The research area has recently focused on local vital signs through immunohistochemistry more than systemic reactions [7].

## 2. Materials and Methods

A systemic review of the literature dealing with the vital reaction of ligature marks in hanging or in ligature strangulation was performed. The review of the literature was carried out according to the PRISMA statement [13]. The research was performed using PubMed, Web of Science, and Scopus with the following keywords: Ligature Mark and Hanging, Ligature Mark and Strangulation, OR Furrow and Hanging OR (noose mark and hanging), Ligature Mark and histology and immunohistochemistry, which were searched in all fields to recognize relevant research available up to 6 October 2021. The search and review of the literature was carried out by two authors independently, and the results were then compared. Inclusion criteria were represented by papers dealing with human ligature marks and immunohistochemistry written in English and published on impacted or indexed scientific journals. Papers not coherent with the aim of the review, case reports, and full text not available were excluded from the research.

The absence of a control group was also considered as an exclusion criteria. Additional exclusion criteria were the following: (1) studies on animal models; (2) without a control group; (3) case reports or case series; (4) full text not available. For duplicate studies, only the article with more detailed information was included.

## 3. Results

**Figure 1:**
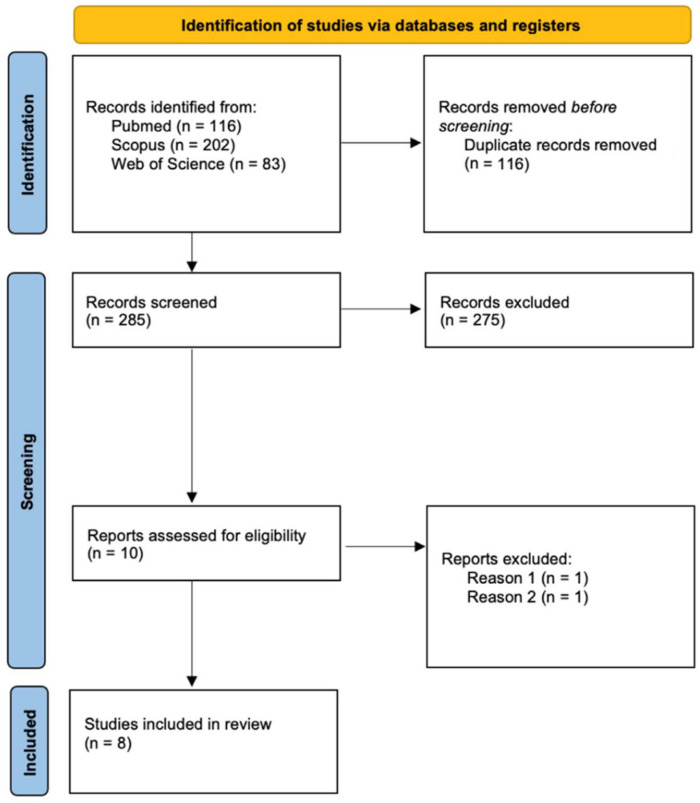
Flowchart depicting the choice of the studies. Exclusion criteria: (1) studies on animal models and (2) without a control group; (3) case reports or case series; and (4) full text not available.

The search of PubMed, Scopus, and Web of Science databases provided a total of 401 articles, and after adjusting for duplicates, 116 studies were discarded. The review of the 285 abstracts resulted in an inclusion of eight papers in the present review. The search provided a total of 285 articles. Once removing all the duplicates (116 studies), the abstracts of 169 articles were reviewed by two of the authors. Only 10 out of 169 studies were coherent with the aim of the review, and they were not case reports or cases series. However, after reviewing the full text of these 10 studies, only eight papers satisfied the inclusion criteria, providing a control group for the immunohistochemical analyses performed (Figure 1 and Table 1).

## 4. Discussion

**Figure 2:**
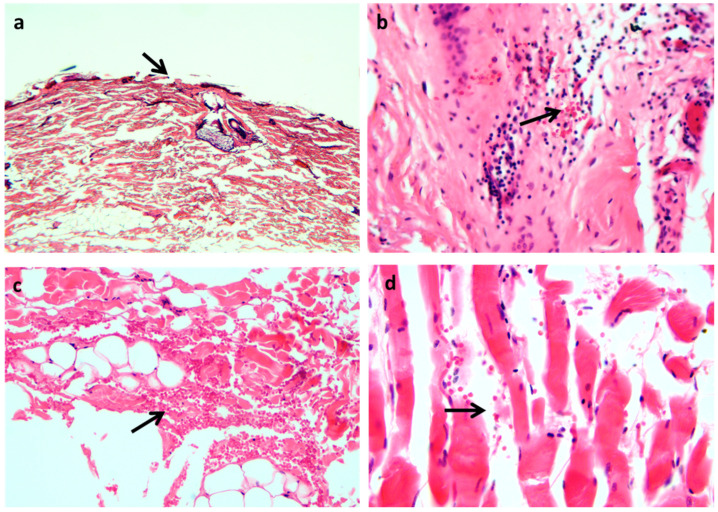
Hanging: In (**a**), the arrow indicates loss of epidermis from trauma (H&E × 10); In (**b**), the arrow indicates an evident inflammatory infiltrate with erythrocyte extravasation in the superficial dermis in correspondence with the sulcus (H&E × 40); In (**c**), the arrow indicates evident erythrocyte extravasation at the dermo-hypodermic border and in the hypodermis (H&E × 40); In (**d**), the arrow indicates clear erythrocyte extravasation into the striated muscle below the sulcus area (H&E × 40). (Images are from the autopsy archive of G.M.).

The results of this systematic review show that (1) only a little research has been carried out on a large series of ligature marks; (2) validated immunohistochemical markers do not yet exist, and (3) the histological morphology of the skin and other organs sampled at autopsy, along with the circumstances of the death investigation, are still crucial for the diagnosis of vitality and age of a skin wound. In fact, the histology of a skin vital lesion, when compared to undamaged vital skin, shows more or less evident abrasions, with loss of the epidermal layer and presence of red blood cells on the surface. In the dermis, the vascular congestion and the erythrocyte extravasation are observed with a focal inflammatory rate that can be useful in dating the time of agony in atypical hangings or in ligature strangulation. The deep dermis, the hypodermis, and the striated muscle portion of the neck region often appear fragmented and associated to evident erythrocyte extravasations as classic signs of trauma. All these microscopic findings are considered signs of vitality and are useful in demonstrating whether the injury was sustained during life and when (Figure 2). The problem occurs when all these aspects appear less evident, such as in ligature marks by soft material, especially in putrefied bodies, and when the mechanism and manner of death are controversial. In these cases, establishing the vitality of sustained injuries is sometimes a challenging task, but systemic vital reactions like those of the circulatory and respiratory systems can be of help. The local vital reaction cannot be independent from the main factors influencing the mechanism of death and the time-course of any wound healing, including the cellular and non-cellular events [18,19].

**Figure 3:**
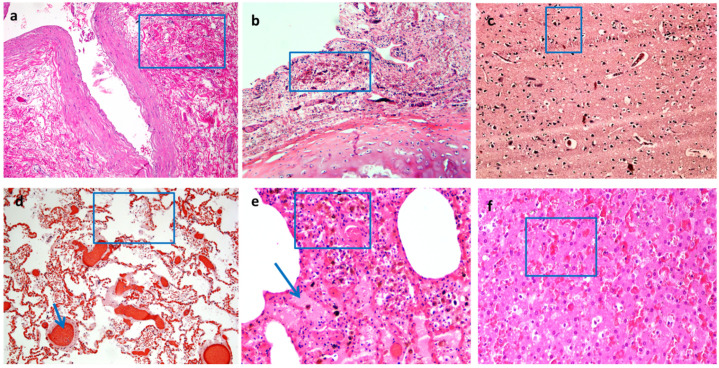
In (**a**), erythrocyte extravasation and soft tissue fragmentation around neck vessels in a case of evident sulcus (blue box) (H&E × 20); In (**b**), laryngeal mucosa site of congestion, erythrocyte extravasation, and initial inflammation of the chorion underlying the loss of lining epithelium, in a case of an absence of a sulcus with agony (blue box) (H&E × 10); In (**c**), hypoxic brain damage (blue box) (H&E × 20); In (**d**), initial rupture of the alveolar septa of the lung (blue box), with emphysema, vascular congestion (blue arrow), and endoalveolar erythrocyte extravasation (H&E × 10); In (**e**), other case of rupture of the alveolar septae of the lungs, edema (blue arrow), and hemorrhage (blue box) (H&E × 20); in (**f**), liver congestion (blue box) (H&E × 20). (Images from the autopsy archive of G.M.).

Visceral congestion is a classical sign in asphyxial deaths commonly associated with cyanosis and capillary hemorrhages (petechiae) often located distant from the site of injury (in the conjunctivae and sclerae or in the visceral pleura and epicardium) due to the intravascular rise of pressure. Along with the multi-organ congestion, common histological findings are erythrocyte extravasations, especially in the parenchymatous organs, but also in the laryngeal tract, with the loss of mucous membranes. Signs of initial neuronal hypoxic degeneration with a white halo or signs of more prolonged hypoxia with a red neuronal appearance may also be observed in the brain. These aspects are fundamental for a differential diagnosis between initial acute hypoxic injury and prolonged hypoxic injury that occurs in chronic hypoxia. Common pulmonary changes are mainly represented by vascular congestion, endo-alveolar erythrocyte extravasations, rupture of the alveolar septa, and emphysema due to mechanical respiratory difficulties and tissue damages produced by the lack of oxygen (Figure 3). The more prolonged is the agony before death, the greater the inflammation will be, mainly at the level of the vital skin wound, such as a ligature mark, but also in the periphery where tissue changes can be the result of a reduced oxygen supply for a prolonged survival interval. Useful information dealing with the mechanism of trauma can be gathered from the histological observation of the peripheral organs, as well as from the exclusive analysis of the vitality of the sulcus itself.

The inflammation process can be assessed based on the lymphocyte populations, as well as of the cytokines. It is a very good marker of the time-course of any wound healing. In fact, there is wide agreement in literature that for every tissue damage, an “inflammation” response is established with almost always the same mechanism [20].

Attention to the microscopic expression of inflammation in the skin samples of hanging have been identified by few studies. The molecules analyzed which have yielded satisfactory results include tryptase, IL15, CD-15, MHC-II, CD1a, AQP1, AQP3, fibronectin, cathepsin D, P-selectin, and IL1beta. Turillazzi et al. (2010) [17] also studied other molecules (TNFα, IL-10, MCP-1, CD 45, CD 4, CD 3, CD 8, CD 68, and CD 20) without reliable results. A very interesting finding was a patchy dermal strong positivity for extracellular tryptase observed immunohistochemically in dermal connective tissue sampled from the marginal zones above and below the hanging marks [17]. The presence of extracellular tryptase at this level is the result of the release of tryptase from mast cells recruited to the site of inflammation. Mast cell degranulation increases tryptase levels in the site of trauma and represents a vital response to the mechanism of neck pressure and abrasion by ligature materials. These findings have been confirmed by other studies of the same research group [16], demonstrating the role of tryptase as a biomarker of vitality. Along with tryptase, CD15 also appears to be another reliable parameter for the determination of ligature marks’ vitality [17]. High levels of CD15 were observed in the superficial dermis of the hanging marks, but no immunohistochemical positivity was found in post-mortem suspension of seven dead bodies. The expression of the CD15 antigen is associated with an IL-15 intense reaction in sub-dermal connective and in perivasal spaces, and this co-expression indicates the role of IL-15 as a proinflammatory cytokine [16] to recruit leukocytes from the lumen of a vessel to a damaged area. IL-15 is a critical regulator of the innate immune response and an inflammatory mediator inducing local neutrophil recruitment in damaged tissues and enhancing phagocytosis, cytoskeleton rearrangement, gene expression, and de novo protein synthesis. IL-15 can also delay apoptosis. In summary, the results of the Italian research group [16,17] demonstrate a strong immunopositivity of tryptase, IL-15, and CD15 in the majority of vital skin wounds. These results have been confirmed by a low expression of the antibodies anti-tryptase, anti-CD15, and anti-FLIP observed in skin samples that are not injured, compared with those found in vital ligature marks [15]. In this study, anti-Troponin I fast skeletal muscle was also tested on muscle samples, resulting in it being hypoexpressed in the not injured muscle samples [15].

The relationship between the vitality of skin wounds and miRNA expression has been investigated by Neri et al. [16]. An over-expression of miR125a-5p and miR125b-5p has been found in skin samples taken from vital lesions. miRNAs are small non-coding RNA molecules that regulate wound inflammation and angiogenesis. Therefore, the expression of specific miRNA undergoes variation during the time-course of healing [21,22].

In the diagnosis of vitality, it is worth of mentioning the role of aquaporins [7,23]. These membrane channel proteins are involved in the transport of water across the cell membrane. They are commonly expressed in epithelial and endothelial cells. Among the aquaporins, AQP3 seems to be a reliable marker of vital neck compression. Ishida Y et al. [2] reported an over-expression of AQP3 in the keratinocytes of compressed skin epidermal layers, such as those of ligature marks. It was assumed that the role of AQP3 is replacement of water loss by evaporation. Therefore, the apical membrane distribution of AQP3 in keratinocytes can enhance after evaporation to replace the water loss [24].

The distribution of MHC class II cells, Langerhans cells, and mast cells in ligature marks has been also investigated immunohistochemically [6,14]. These cells play a crucial role in the early response to tissue damage. The results show that dendritic cells CD1a+ are mostly present in vital ligature marks, along with an over-expression of the INOS [6,14].

There are also other markers that could be considered indicative signs of wounds vitality. The expression of Fibronectin, Cathepsin D, and P-selectin was analyzed in two different experimental studies [4,25]. Fibronectin is a high-molecular weight glycoprotein involved in processes of cell adhesion and cell migration, re-epithelialization, and extracellular matrix (ECM) formation to promote wound healing. Cathepsin D is a proteinase that has been regarded as an important biomarker of wound vitality, but a high expression level of this enzyme has been found both in vital and post-mortem injuries. P-selectin is a transmembrane protein present on the surfaces of activated endothelial cells and activated platelets useful to estimate wound age in injuries with a short survival time. The results of the immunohistochemical studies by Legaz et al. [4] showed a very strong positive reaction of Fibronectin in the derma of the vital ligature marks, while in the basal layer of the epidermis, an increase of the granular staining pattern, characteristic of Cathepsin D levels, and a weak positive immunoreactivity of P-selectin expression have been found. Strongly positive Fibronectin complexes were detected in not-recent skin wounds of 13 and 30 days old. However, the reliability of these enzymatic markers as vital signs is controversial, because these proteins are not specific to vital injuries [26,27]. In Table 2, the main characteristics and function of the immunohistochemical markers investigated by the selected articles have been summarized.

The aim of this systematic review was to detect how useful the immunohistochemical markers can be in the assessment of the vitality of the ligature marks. Based on our results, the application of immunohistochemistry for the age estimation and vitality of skin wounds can be useful but still challenging. Caution is also needed in the interpretation of the results. Several factors can affect the reliability of immunohistochemical results, like the supra-vital reactions and the post-mortal changes. False negatives and false positives can occur in immunohistochemical reactions due to inadequate fixation of tissue samples and insufficient antibody incubation because of cross-reactivity. Unfortunately, it is still a long and winding road to get validated immunohistochemical markers for a reliable diagnosis in order to distinguish, with certainty, vital ligature marks from skin lesions that occurred after death [4]. The efforts of the researchers are still very few, and data must necessarily be validated by standardized experiments on larger series of the study groups. In particular, there is a strong need for methodological standardization in research studies and validation protocols to promote consistency and comparability among studies.

In the future, immunohistochemical methods and/or molecular biology can be a comfort to histological diagnosis. At the base of any damage inflicted in life, the key to understanding the mechanisms of vital reaction is still inflammation [20]. The most important signs of a vital reaction are still those of inflammation and early chemical mediators, especially when it is necessary to estimate age and vitality of a skin wound like a ligature mark.

## 5. Conclusions

The assessment of the vitality and age of a ligature mark is still a challenging topic in forensic pathology. One immunohistochemical biomarker alone is of limited value, as it would be studied according to all systemic vital reactions, like those of the circulatory and respiratory systems, and in the light of all factors that can affect the reliability of immunohistochemistry.

The state of art in the vitality of skin wounds shows that most of the relevant markers can be successfully observed by traditional histology (H&E staining). They are still the most reliable morphological criteria for assessing the vital reaction and for estimating wound age. In the future, immunohistochemical markers could be of use to basic diagnosis, and we look forward tto new tools to determine whether a human skin wound has been sustained during life or not, especially in cases where the mechanism and mode of death are controversial.

Validation protocols to promote consistency and comparability among immunohistochemical markers and to improve their reliability in routine diagnostics of vital reaction are still needed.

## Figures and Tables

**Table 1:** Summary of the items recorded from the eight papers included in the study. Legend: hematoxylin and eosin (H&E); immunohistochemistry/immunofluorescence (IH/IF); documented (√); not documented (n.d.); CD1a (dendritic cells and Langerhans cells antigen), INOS (isozyme nitric oxide synthases), MHC-II (major histocompatibility complex-class II), UEA (Ulex Europaeus Agglutinin), Spm250 (granulocytes antigen); dendritically cells (DL); Langerhans cells (LC); mast cells (MC); antiapoptotic protein (FLIP), leukocyte marker-granulocytes (CD15); aquaporins (AQP1, AQP3); tumor necrosis factor (TNFα); interleukin (IL); Monocyte Chemoattractant Protein-1 (MCP-1); T-cell lymphocytes (CD4, CD3, CD 8); common leukocyte antigen (CD45); histiocytic marker (CD68); B-cell lymphocytes (CD20).

Reference	Ligature Marks (Skin Sample n.)	Control Group (Skin Sample n.)	H&E	IH/IF (Abs)	Results
Focardi M. et al., 2021 [14]	10 cases of suicidal hanging	10 samples taken 20 cm below the ligature marks; 10 vital lesions (surgical wounds, abrasions, and lacerations of the knee or ankles); 10 samples of post-mortem wounds	√	CD1a, iNOS, MHC-II, UEA, Spm250.	The inflammatory infiltrate is greater in vital wounds and in hanging ligature than in controls and post-mortem injuries. Dendritic cells in the spinous or granular layers of the epidermis in hanging marks were similar to vital wounds. Langerhans cells express iNOS more in the hanging grooves than other groups of lesions; while the percentage of these cells that do not express the enzyme is similar to the hanging marks and vital wounds, it is lower in the controls and higher in post-mortal lesions. The keratinocytes’ expression of iNOS is similar in all groups of lesions. Mast cells express iNOS in vital wounds in very few cellular types, but in hanging furrows, the expression of iNOS characterizes a large fraction of the Mast cells population.
Maiese et al., 2020 [15]	21 cases of suicidal hanging	13 skin samples taken from victims of opioid overdoses, car accidents, sudden cardiac death, and post-mortem suspension of bodies	√	FLIP (ab8421), Tryptase, CD15.	Flattening of the epidermal layers with liquid-filled vesicles; mild leukocyte reactions. Intracytoplasmic depletion of FLIP in the epidermal layers from hanging.
Focardi M. et al., 2020 [6]	20 cases of suicidal hanging (10 from superficial layers and 10 from deep layers of the skin)	10 samples of vital wounds, abrasions and lacerations 10 samples of post-mortem wounds	√	MHC-II, CD1a	Different expression of MHC class II from dendritic cells, Langerhans cells, and macrophages in the skin 20 cm far from the wound; in the vital wound; in the ligature mark, and in post-mortem lesion. Dendritic cells CD1a+ is mostly present in vital lesions and hanging marks.
Neri M. et al., 2019 [16]	36 ligature marks	28 samples of the not-injured skin of the neck	√	Tryptase, IL-15, andCD15.	CD15 (neutrophils), tryptase (mast cells), and IL-15 are strongly positive in the marginal zones above and below the hanging marks. IL-15 positivity is in the dermis and around the vessels. CD15 and tryptase are intensely positive in the dermal connective tissue. CD15 and IL-15 are co-expressed and, there is increased expression of miR125a-5p and miR125b-5p.
Ishida Y. et al., 2018 [2]	35 cases of hanging; 21 cases of ligature strangulation	Samples of not-injured skin from the same corpse	n.d.	AQP1 and AQP3	The AQP3 expression on keratinocytes is enhanced in ligature marks.
Legaz I. et al., 2018 [4]	15 cases of suicidal hanging	15 samples of not-injured skin from the same corpses	n.d.	Fibronectin, Cathepsin D, and P-selectin	Fibronectin is strongly positive in the derma of ligature marks. Cathepsin D expression is increased in the basal layer of the epidermis in ligature marks. P-selectin expression is decreased in the basal layer of the epidermis in ligature marks.
Turillazzi E. et al., 2010 [17]	49 cases of suicidal hanging	14 samples of not-injured skin of the neck; 7 samples of post-mortem hanging	√	Tryptase, Fibronectin, TNFα, IL-6, IL-8, IL-10, IL-15, IL-1ß, MCP-1, CD45, CD4, CD3, CD8, CD68, CD20, and CD15.	CD15, tryptase, and IL-15 are strongly positive in the dermal of marginal zones above and below the hanging marks. IL-15 is located around the dermal vessels and is diffusely sparse in depth. CD15 and tryptase positivity is in dermal connective tissue. CD15 and IL- 15 are co-expressed.

**Table 2:** Markers immunohistochemically investigated in the literature: role, characteristics, and identification. isozyme Nitric Oxide Synthases (iNOS); Aquaporins (AQP); interleukin (IL).

Marker	Role, Characteristics and Cell Type Expression
CD1A	It is a membrane glycoprotein related to the MHC that mediates the presentation of antigens to T cells. It is expressed by thymocytes, dendritic cells, and Langerhans cells.
iNOS	It is an isozyme synthase involved in the immune response by binding calmodulin and producing NO (nitric oxide). It is induced in different cell types of the cardiovascular system like endothelial cells and in immune cells.
FLIP	It is an anti-apoptotic protein produced by different cell types. It regulates the activity of caspase-8 and modulates the apoptotic signal mediated by TRAIL-R1/R2 (death receptors), TNFR1 (TNF receptor), and Toll-like receptors.
Tryptase	It is a serine protease present in the granules of the cytoplasm of mast cells; it is implicated in hypersensitivity reactions and anaphylaxis.
AQP1/AQP3	The aquaporins (AQPs) are a family of channel proteins involved in the passage of water through the cells. They are predominantly expressed by the epithelium of the proximal renal tubules, erythrocytes (AQPs), and visceral pleura epithelium (AQP1) and by the epithelium of the trachea, bronchi, and nasopharynx (AQP3).
Fibronectin	It is a glycoprotein of the extracellular matrix (connective tissue) that plays a fundamental role in the repair of tissue damage. It is produced by many cell types.
Cathepsin D	It is a lysosomal aspartyl protease which degrades fibronectin and laminin. It is present in cell lysosomes and involved in the pathogenesis of several diseases such as breast cancer and possibly Alzheimer disease.
P-selectin	It is a membrane protein that acts as a cell adhesion molecule on the surfaces of endothelial cells and activated platelets.
IL-15	It is a cytokine mainly produced by macrophages. It promotes cell survival of natural killer (NK) lymphocytes and activates the differentiation of NK lymphocytes.
CD15	It is an adhesion molecule produced by granulocytes implicated in phagocytosis and chemotaxis.

## References

[1] Di Maio V.J.M., Di Maio D.J. (2001). Forensic Pathology.

[2] Ishida Y., Kuninaka Y., Nosaka M., Shimada E., Hata S., Yamamoto H., Hashizume Y., Kimura A., Furukawa F., Kondo T. (2018). Forensic application of epidermal AQP3 expression to determination of wound vitality in human compressed neck skin. Int. J. Leg. Med..

[3] Dettmeyer R.B., Verhoff M.A., Schutz H.F. (2014). Forensic Medicine.

[4] Legaz I., Pérez-Cárceles M.P., Gimenez M., Martínez-Díaz F., Osuna E., Luna A. (2018). Immunohistochemistry as a tool to characterize human skin wounds of hanging marks. Rom. J. Leg. Med..

[5] Langlois N.E., Gresham G.A. (1991). The ageing of bruises: A review and study of the colour changes with time. Forensic Sci. Int..

[6] Focardi M., Puliti E., Grifoni R., Palandri M., Bugelli V., Pinchi V., Norelli G.A., Bacci S. (2020). Immunohistochemical localization of Langerhans cells as a tool for vitality in hanging mark wounds: A pilot study. Aust. J. Forensic Sci..

[7] Madea B., Doberentz E., Jackowski C. (2019). Vital reactions—An updated overview. Forensic Sci. Int..

[8] Sauvageau A., Racette S. (2007). Agonal sequences in a filmed suicidal hanging: Analysis of respiratory and movement responses to asphyxia by hanging. J. Forensic Sci..

[9] Cecchi R. (2010). Estimating wound age: Looking into the future. Int. J. Leg. Med..

[10] Dettmeyer R.B. (2018). Forensic Histopathology.

[11] Campobasso C.P., Colonna M.F., Zotti F., Sblano S., Dell’Erba A.S. (2012). An immunohistochemical study of pulmonary surfactant apoprotein A (SP-A) in forensic autopsy materials. Rom. J. Leg. Med..

[12] Sblano S., Campobasso C.P., Zotti F., Arpaio A., Di Vella G., Colonna M.F. (2012). Beta-APP immunoreactivity as diagnostic tool of diffuse axonal injury (DAI). Rom. J. Leg. Med..

[13] Page M.J., McKenzie J.E., Bossuyt P.M., Boutron I., Hoffmann T.C., Mulrow C.D., Shamseer L., Tetzlaff J.M., Akl E.A., Brennan S.E. (2021). The PRISMA 2020 statement: An updated guideline for reporting systematic reviews. BMJ.

[14] Focardi M., Bugelli V., Venturini M., Bianchi I., Defraia B., Pinchi V., Bacci S. (2020). Increased expression of iNOS by Langerhans cells in hanging marks. Aust. J. Forensic Sci..

[15] Maiese A., De Matteis A., Bolino G., Turillazzi E., Frati P., Fineschi V. (2020). Hypo-Expression of Flice-Inhibitory Protein and Activation of the Caspase-8 Apoptotic Pathways in the Death-Inducing Signaling Complex Due to Ischemia Induced by the Compression of the Asphyxiogenic Tool on the Skin in Hanging Cases. Diagnostics.

[16] Neri M., Fabbri M., D’Errico S., Di Paolo M., Frati P., Gaudio R.M., La Russa R., Maiese A., Marti M., Pinchi E. (2019). Regulation of miRNAs as new tool for cutaneous vitality lesions demonstration in ligature marks in deaths by hanging. Sci. Rep..

[17] Turillazzi E., Vacchiano G., Luna-Maldonado A., Neri M., Pomara C., Rabozzi R., Riezzo I., Fineschi V. (2010). Tryptase, CD-15 and IL-15 as reliable markers for the determination of soft and hard ligature marks vitality. Histol. Histopathol..

[18] Mansueto G., Di Napoli M., Mascolo P., Carfora A., Zangani P., Della Pietra B., Campobasso C.P. (2021). Electrocution Stigmas in Organ Damage: The Pathological Marks. Diagnostics.

[19] Buonomo R., Giacco F., Vasaturo A., Caserta S., Guido S., Pagliara V., Garbi C., Mansueto G., Cassese A., Perruolo G. (2012). PED/PEA-15 controls fibroblast motility and wound closure by ERK1/2-dependent mechanisms. J. Cell. Physiol..

[20] Mansueto G., Costa D., Capasso E., Varavallo F., Brunitto G., Caserta R., Esposito S., Niola M., Sardu C., Marfella R. (2019). The dating of thrombus organization in cases of pulmonary embolism: An autopsy study. BMC Cardiovasc. Disord..

[21] Sen C.K., Roy S. (2012). OxymiRs in cutaneous development, wound repair and regeneration. Semin. Cell Dev. Biol..

[22] Banerjee J., Sen C.K. (2015). MicroRNA and Wound Healing. Adv. Exp. Med. Biol..

[23] Betz P. (1995). Immunohistochemical parameters for the age estimation of human skin wounds. Am. J. Forensic Med. Pathol..

[24] Cao C., Wan S., Jiang Q., Amaral A., Lu S., Hu G., Bi Z., Kouttab N., Chu W., Wan Y. (2008). All-trans retinoic acid attenuates ultraviolet radiation-induced down-regulation of aquaporin-3 and water permeability in human keratinocytes. J. Cell. Physiol..

[25] Legaz Pérez I., Falcón M., Gimenez M., Diaz F.M., Pérez-Cárceles M.D., Osuna E., Nuno-Vieira D., Luna A. (2017). Diagnosis of Vitality in Skin Wounds in the Ligature Marks Resulting from Suicide Hanging. Am. J. Forensic Med. Pathol..

[26] Grellner W., Madea B. (2007). Demands on scientific studies: Vitality of wounds and wound age estimation. Forensic Sci. Int..

[27] Casse J.M., Martrille L., Vignaud J.M., Gauchotte G. (2016). Skin wounds vitality markers in forensic pathology: An updated review. Med. Sci. Law.

